# MicroRNAs recruit eIF4E2 to repress translation of target mRNAs

**DOI:** 10.1007/s13238-017-0444-0

**Published:** 2017-07-28

**Authors:** Shaohong Chen, Guangxia Gao

**Affiliations:** 1 CAS Key Laboratory of Infection and Immunity, CAS Center for Excellence in Biomacromolecules, Institute of Biophysics, Chinese Academy of Sciences, Beijing, 100101 China; 2 University of Chinese Academy of Sciences, Beijing, 100101 China

**Keywords:** microRNAs, translation repression, 5′ cap, eIF4E2, TNRC6A

## Abstract

MicroRNAs (miRNAs) recruit the RNA-induced silencing complex (RISC) to repress the translation of target mRNAs. While the 5′ 7-methylguanosine cap of target mRNAs has been well known to be important for miRNA repression, the underlying mechanism is not clear. Here we show that TNRC6A interacts with eIF4E2, a homologue of eIF4E that can bind to the cap but cannot interact with eIF4G to initiate translation, to inhibit the translation of target mRNAs. Downregulation of eIF4E2 relieved miRNA repression of reporter expression. Moreover, eIF4E2 downregulation increased the protein levels of endogenous IMP1, PTEN and PDCD4, whose expression are repressed by endogenous miRNAs. We further provide evidence showing that miRNA enhances eIF4E2 association with the target mRNA. We propose that miRNAs recruit eIF4E2 to compete with eIF4E to repress mRNA translation.

**Electronic supplementary material:**

The online version of this article (doi:10.1007/s13238-017-0444-0) contains supplementary material, which is available to authorized users.

## Introduction

MicroRNAs (miRNAs) are 21–23 nucleotides noncoding RNAs that regulate the expression of messenger RNAs. miRNA-mediated gene silencing plays important roles in a variety of biological processes (Flynt and Lai, 2008; Jonas and Izaurralde, 2015). miRNAs in complex with Argonaute proteins (Ago1–4) bind to target mRNAs through nucleotide pairing and recruit one of the TNRC6 proteins (TNRC6A-6C) to form the RNA-induced silencing complex (RISC) (Pfaff and Meister, 2013). RISC can promote target mRNA translation repression, degradation or both (Fabian et al., 2010).

There is increasing evidence indicating that translation initiation is a major target of miRNA repression (Pillai et al., 2005; Meister, 2007; Mathonnet et al., 2007; Fukaya et al., 2014; Humphreys et al., 2005; Thermann and Hentze, 2007; Fukaya and Tomari, 2012; Wang et al., 2008; Ricci et al., 2013; Gu et al., 2014). Cellular mRNAs have a 7-methylguanosine (m7GpppN) cap structure at the 5′ end, which promotes translation and mRNA stability (Varani, 1997). The cap is recognized by the translation initiation factor eIF4E, which recruits other translation initiation factors, including eIF4G, the RNA helicase eIF4A, eIF2, eIF3 and the 40S small ribosomal subunit to initiate translation (Gingras et al., 1999). eIF4A and its homologue eIF4A2 are important for unwinding structures in the 5′ untranslated regions (5′UTRs) (Lu, et al., 2014). It was reported that RISC-induced dissociation of eIF4A or eIF4A2 caused miRNA repression (Lu et al., 2014; Meijer et al., 2013; Fukao et al., 2014). These results partially explain how miRNAs repress translation initiation.

It has been well documented that the 5′ cap is important for miRNA repression. In contrast to the cap-containing mRNA reporters, internal ribosome entry site (IRES)-initiated mRNA reporters are refractory to miRNA silencing (Pillai et al., 2005; Mathonnet et al., 2007; Humphreys et al., 2005; Walters et al., 2010). IRES can bypass the cap to recruit ribosomes to the mRNA internally to initiate translation (Jackson et al., 2010). Addition of a cap to the IRES mRNA reporters conferred sensitivity to miRNA silencing (Walters et al., 2010). On the other hand, some other work showed that IRES-dependent translation can also be repressed by miRNA (Humphreys et al., 2005; Petersen et al., 2006). As is a proper explanation in Valencia-Sanchez et al.’s 2006 review, miRNAs also work through other elements to repress target mRNA, such as poly(A) tail and 5′ cap structure and these miRNA-repression related elements might be missing in some reporters.

That cap optimizes miRNA silencing strongly suggests that a cap-binding protein should be involved in miRNA repression (Kiriakidou et al., 2007). It was reported that human Ago2 bound to the cap through two conserved phenylalanine residues (F450 and F505) and thereby prevented the recruitment of eIF4E in miRNA repression (Kiriakidou et al., 2007). However, this conclusion was challenged by later studies. Those two residues were shown to be important for Ago interaction with GW182 rather than binding to the cap in both human and flies (Eulalio et al., 2008). Further structure analysis showed that in human Ago2 one of the aromatic side chains F450 is buried in the hydrophobic core (Kinch and Grishin, 2009) and MID domain of human Ago2 cannot bind cap analogues significantly (Frank et al., 2011), although Drosophila Ago1 and Neurospora Argonaute contains a 5′ cap binding site (Boland et al., 2010; Djuranovic et al., 2010). Moreover, fly Ago-RISC associated with target mRNA in a manner independent of GW182 and repressed translation without affecting eIF4E recognition of the cap (Fukaya et al., 2014), further arguing against the idea that Ago binding to the cap is required for translation repression.

eIF4E2 is a homologue of eIF4E (Rom et al., 1998). The binding affinity of eIF4E2 for the cap is about 100-fold lower than eIF4E, and the abundance in cells is about 10-fold lower (Zuberek et al., 2007). In addition, unlike eIF4E, eIF4E2 does not interact with eIF4G to initiate translation (Rom et al., 1998). When recruited to the cap of an mRNA by transacting factors, eIF4E2 can repress the translation of the target mRNA. For example, Drosophila Bicoid recruits eIF4E2 to suppress caudal translation (Cho et al., 2005), and TTP recruits eIF4E2 to repress the translation of AU-rich element-containing mRNAs (Tao and Gao, 2015; Fu et al., 2016).

eIF4E2 was reported to interact with 4E-T (Kubacka et al., 2013), which participates in translation repression and mRNA decay in TTP or microRNA mediated silencing (Kamenska et al., 2014; Kamenska et al., 2016; Nishimura et al., 2015). A very recent research shows that eIF4E2 effects miRNA mediated translation silencing by competing with eIF4E for binding 4E-T (Chapat, 2017). In the present study, we confirmed that eIF4E2 is required for miRNA mediated translation repression. In addition, we provide evidence indicating that TNRC6A, the core component of RISC, can directly recruit eIF4E2 to target mRNA to repress translation.

## Results

### The 5′ cap of target mRNA is required for optimal miRNA-mediated gene silencing

**Figure 1:**
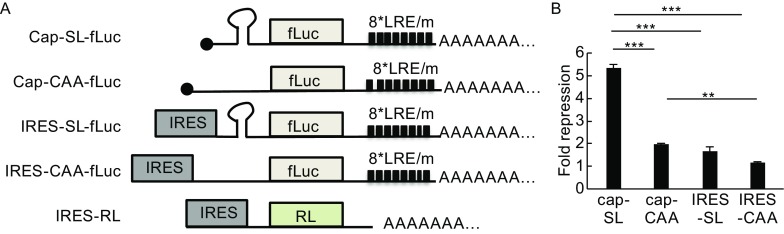
The cap structure of target mRNA is required for optimal miRNA-mediated gene silencing. (A) Schematic representation of the reporter mRNAs. Eight tandem Let-7a response elements (LRE) or mutants (REm) were cloned downstream of the coding sequence of firefly luciferase (fLuc). A stem-loop (SL) structured fragment or a fragment containing (CAA)_18_ was inserted into the 5′UTR of the reporters indicated. (B) Reporter mRNAs were transfected into HeLa cells. A control reporter expressing renilla luciferase (RL) driven by EMCV IRES was included. At 24 h postinfection, luciferase activities were measured and firefly luciferase activity was normalized with renilla luciferase activity. Data presented are means ± SD of three independent experiments. The *P* value is determined by two-tailed Student’s *t* test. ns, nonsignificant. **P < 0.01; ***P < 0.005

To determine the contributions of the cap and structured 5′UTR to miRNA silencing, reporter mRNAs expressing firefly luciferase (fLuc) were generated by *in vitro* transcription (Fig. 1A). In the 3′UTR of these reporters, there were eight tandem Let7a-responsive elements (LREs), which are responsive to endogenous let-7a in HeLa cells. Reporters containing mutant responsive elements (REm) were constructed to serve as negative controls (Fig. 1A). In the 5′UTR there was a stem-loop (SL), which mimics a secondary structure, or repetitive CAA sequences, which cannot form secondary structures. The translation of the reporters was driven either by the cap or by EMCV IRES. EMCV IRES-driven translation does not need eIF4E but requires the rest translation initiation factors (Jackson et al., 2010). A reporter expressing renilla luciferase (RL) driven by EMCV IRES was used to serve as a control for transfection efficiency and sample handling (Fig. 1A). The reporters were transfected into HeLa cells and luciferase activities were measured. The response of a reporter to let-7a silencing was indicated by fold repression, which was calculated as the luciferase activity expressed from the REm reporter divided by that expressed from the LRE reporter. That equivalent amounts of the mRNA reporters were transfected into the cells was confirmed by RT-qPCR (Fig. S1). Data showed that let-7a repressed the expression of the reporter containing both the cap and the stem-loop (Cap-SL-fLuc) by about 5-fold (Fig. 1B). The reporter containing the stem-loop and not the cap (IRES-SL-fLuc) was repressed by about 2-fold (Fig. 1B). The reporter containing the cap but not the stem-loop (Cap-CAA-fLuc) was still responsive, with the repression about 2-fold (Fig. 1B). When both the cap and the stem-loop were removed, the reporter (IRES-CAA-fLuc) was barely responsive (Fig. 1B). These results confirmed that both the cap and a structured 5′UTR contribute to miRNA repression.

### TNRC6A interacts with eIF4E2

**Figure 2:**
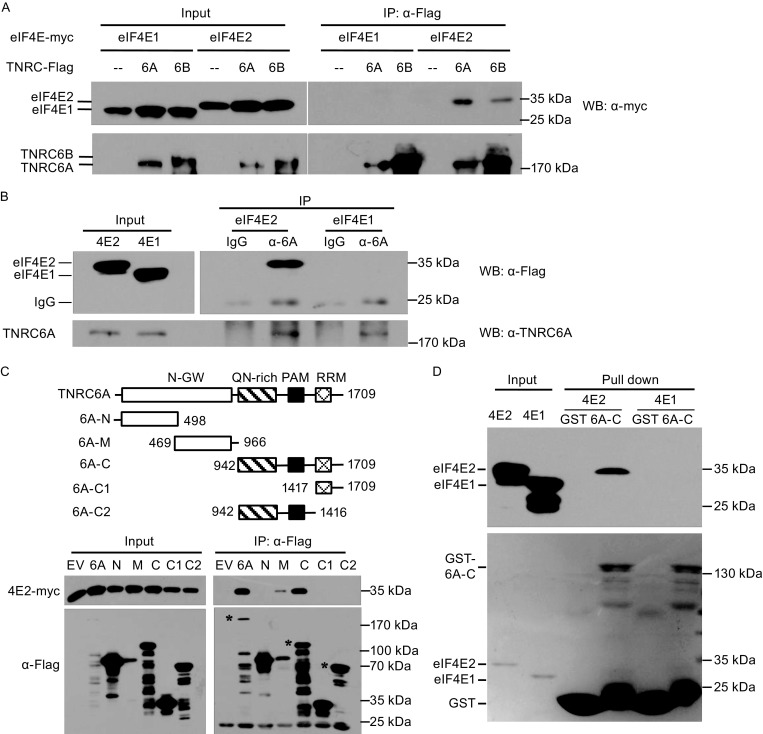
TNRC6A interacts with eIF4E2. (A) Plasmids expressing proteins indicated were transfected into HEK293T cells. At 48 h posttransfection, cells were lysed and the lysates were immunoprecipitated (IP) in the presence of RNase A. The precipitates were resolved on SDS-PAGE followed by Western blotting. (B) A plasmid expressing Flag-tagged eIF4E1 or eIF4E2 was transfected into HEK293T cells. At 48 h posttransfection, cells were lysed and the lysates were immunoprecipitated with the anti-TNRC6A antibody or control IgG in the presence of RNase A. The precipitates were resolved on SDS-PAGE followed by Western blotting. (C) Upper: schematic representation of TNRC6A truncation mutants. Lower: A plasmid expressing the TNRC6A mutant indicated and a plasmid expressing myc-tagged eIF4E2 were transiently transfected into HEK293T cells. At 48 h posttransfection, cells were lysed and the lysates were immunoprecipitated with anti-Flag antibody in the presence of RNase A followed by Western blotting. (D) Bacterially expressed Flag-tagged eIF4E2 or eIF4E1 was incubated with Glutathione Sepharose 4B bound fusion protein of GST and the C-terminal domain of TNRC6A (6A-C). The precipitates were washed and resolved on SDS-PAGE followed by commassie brilliant blue staining (lower) and Western blotting (upper)

We next explored the interactions between RISC and cap-binding proteins eIF4E (referred to as eIF4E1 hereafter) and its homologue eIF4E2. TNRC6 proteins, key players in miRNA repression, were tested for their interactions with eIF4E1 and eIF4E2 by coimmunoprecipitation assays. Data showed that immunoprecipitation of both TNRC6A and 6B coprecipitated eIF4E2 (Fig. 2A). In contrast, immunoprecipitation of TNRC6A or TNRC6B failed to coprecipitate eIF4E1 (Fig. 2A). The interaction between TNRC6A/6B and 4E2 is consistent with the BioID results in the recent paper by Chapat et al. (2017). We tried to detect the interaction between endogenous TNRC6A and eIF4E2 but failed (data not shown). This could be accounted for by technical difficulties such as low abundance of the endogenous eIF4E2, degradation of TNRC6A, and lack of good antibodies. We then analyzed the interaction between endogenous TNRC6A and overexpressed eIF4E2. Data showed that immunoprecipitation of endogenous TNRC6A coprecipitated Flag-tagged eIF4E2 but not eIF4E1 (Fig. 2B).

To map the domain(s) of TNRC6A that is involved in its interaction with eIF4E2, TNRC6A truncation mutants were constructed and tested for their interactions with Flag-tagged eIF4E2 (Fig. 2C). Data showed that the C-terminal domain of TNRC6A (TNRC6A-C) displayed considerable interaction with eIF4E2 while the other domains failed to do so although the middle domain (TNRC6A-M) had some weak interaction (Fig. 2C). Further truncations of TNRC6A-C abolished its interaction with eIF4E2 (Fig. 2C). To test whether the interaction between TNRC6A-C and eIF4E2 is direct, they were bacterially expressed, partially purified and analyzed by the pull-down assays. Indeed, the recombinant GST-tagged TNRC6A-C pulled down eIF4E2 but not eIF4E1 (Fig. 2D). These results indicate that eIF4E2 directly interacts with the C-terminal domain of TNRC6A.

### Downregulation of eIF4E2 impairs miRNA silencing

**Figure 3:**
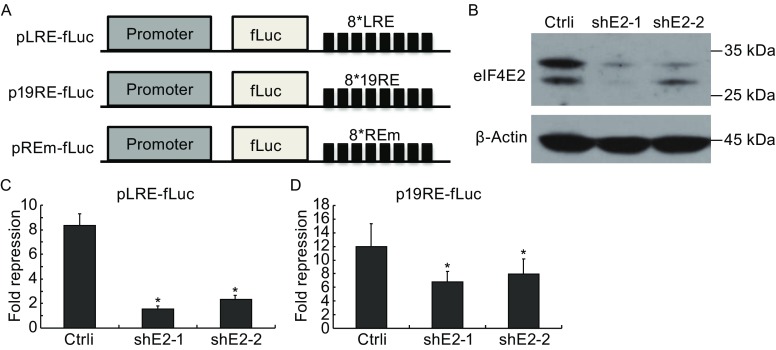
Downregulation of eIF4E2 impairs miRNA repression of reporter expression. (A) Schematic representation of reporter plasmids expressing firefly luciferase (fLuc). Eight tandem Let-7a response elements (LRE), miR-19 response elements (19RE), or the mutant response elements (REm) were cloned downstream of the coding sequence of firefly luciferase. (B) Plasmids expressing shRNAs targeting two different sites of eIF4E2 (shE2-1 and shE2-2) were individually transfected into HEK293 cells. At 48 h postinfection, cells were harvested and the lysates were resolved by SDS-PAGE followed by Western blotting. (C) The pLRE-fLuc reporter and a plasmid expressing a control shRNA or the shRNA targeting eIF4E2 were transfected into HEK293 cells with or without a plasmid expressing Let7a. A renilla luciferase-expressing control reporter was included. At 48 h postinfection, luciferase activities were measured. Firefly luciferase activity was normalized with renilla luciferase activity. Fold repression was calculated as the normalized luciferase activity in the absence of Let7a divided by that in the presence of Let7a. Data presented are means ± SD of three independent experiments. (D) p19RE-fLuc or pREm-fLuc was transfected into HEK293 cells together with a plasmid expressing a control shRNA or the shRNA targeting eIF4E2. A renilla luciferase-expressing reporter was included to serve as a control. At 48 h postinfection, luciferase activities were measured. Firefly luciferase activity was normalized with renilla luciferase activity. Fold repression was calculated as normalized luciferase activity expressed from pREm-fLuc divided by that expressed from p19RE-fLuc. Data presented are means ± SD of three independent experiments. The *P* value is determined by two-tailed Student’s *t* test. *P < 0.05

To explore the role of eIF4E2 in miRNA-mediated translational repression, we analyzed the effects of eIF4E2 downregulation on miRNA silencing of two reporters, the Let7a-responsive LRE reporter and the miR-19-responsive 19RE reporter (Fig. 3A). Two shRNAs targeting different sites of eIF4E2 were constructed and confirmed for their ability to downregulate the expression of endogenous eIF4E2 (Fig. 3B). In the absence of the shRNA targeting eIF4E2, ectopic expression of let-7a inhibited the LRE reporter expression by about 8-fold (Fig. 3C). In comparison, downregulation of eIF4E2 significantly reduced the repression (Fig. 3C). The relatively high expression level of miR-19 in HEK293 cells allowed us to use the endogenous miR-19 to inhibit 19RE reporter expression. A reporter containing REm (Fig. 3A) was used as a negative control. MiR-19 repression of the 19RE reporter expression was defined as the luciferase activity expressed from the REm reporter divided by that expressed from the 19RE reporter. In the absence of the shRNA targeting eIF4E2, the repression was about 12-fold (Fig. 3D). In comparison, downregulation of eIF4E2 reduced the repression to about 6-fold (Fig. 3D). These results indicate that downregulation of eIF4E2 impaired miRNA repression of reporter expression.

### Downregulation of eIF4E2 relieves miRNA repression of target mRNA translation

**Figure 4:**
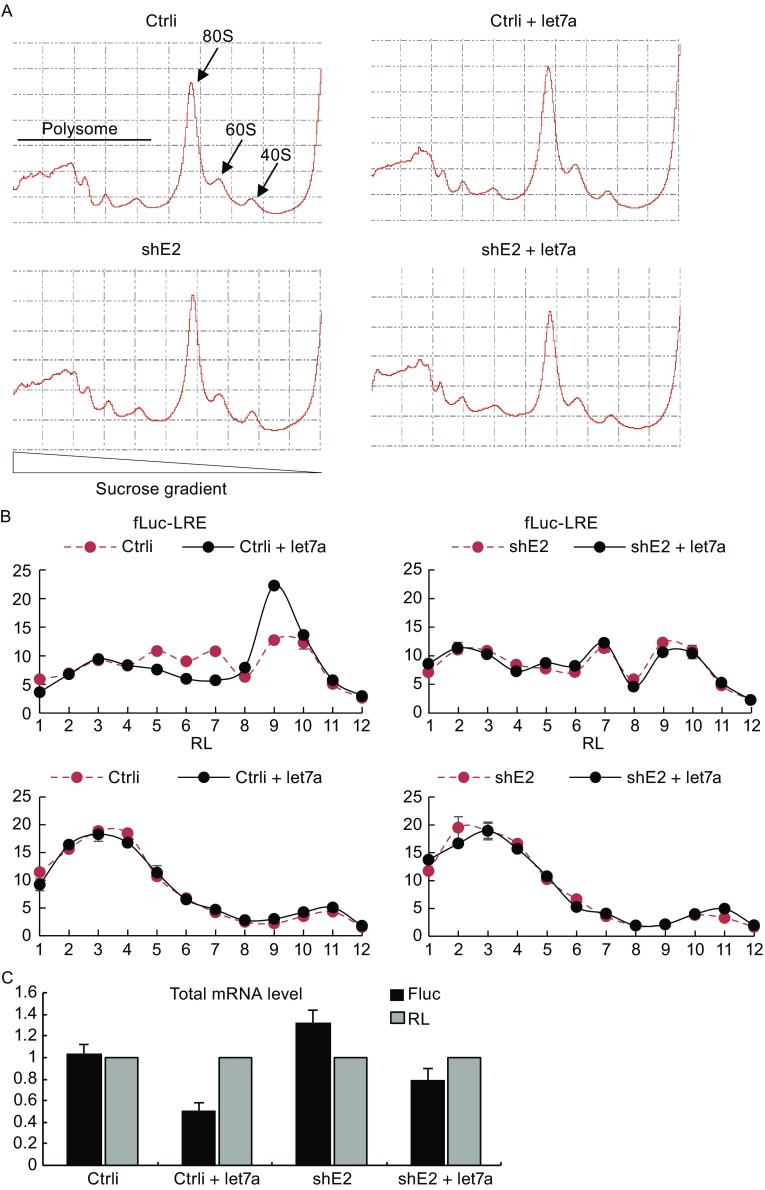
Downregulation of eIF4E2 relieves miRNA repression of target mRNA translation. (A) A control shRNA (Ctrli) or an shRNA targeting eIF4E2 (shE2) were transfected into HEK293 cells with pLRE-fLuc reporter and the control reporter phRL-CMV, together with a plasmid expressing let-7a or a control miRNA. At 48 h posttransfection, the cells were lysed with RNC buffer containing cycloheximide. The lysate was clarified and separated by velocity sedimentation in a sucrose continuous gradient. Fractions were collected and polysome profile was plotted by 254-nm absorption value. (B) fLuc and RL reporter mRNA levels in each fraction were quantified by RT-qPCR. Y-axis represents the percentage of mRNA in each fraction out of the total mRNA in all the fractions. (C) The total mRNA input before ultra-centrifugation and fractioning

We next used the polysome profiling assay to confirm that eIF4E2 participates in miRNA mediated translational repression. Overexpression of miRNA let-7a or downregulation of eIF4E2 did not affect the pattern of global mRNA translation (Fig. 4A). Let-7a overexpression reduced the percentage of fLuc-LRE reporter mRNA in the polysome fractions, which indicates that miRNA-mediated repression targets the translational process (Pillai et al., 2005). Downregulation of eIF4E2 relieved the repression, while the control RL mRNA distribution was not affected (Fig. 4B). Notably, let-7a overexpression reduced the total mRNA level of fLuc-LRE but not the control reporter RL, while downregulation of eIF4E2 did not affect the total mRNA level of either reporter (Fig. 4C), indicating that eIF4E2 mainly plays a role in the process of miRNA mediated translational repression but not the process of RNA decay.

### Downregulation of eIF4E2 increases the translational efficiency of endogenous IMP1

**Figure 5:**
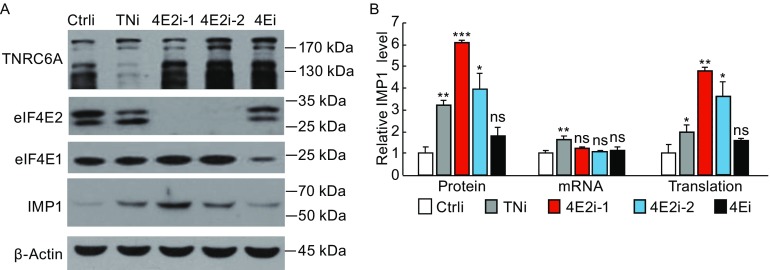
Downregulation of eIF4E2 increases the protein levels of endogenous IMP1. HeLa cells were transfected with siRNAs indicated. At 48 h posttransfection, cells were lysed. (A) A fraction of the lysate was subjected to SDS-PAGE followed by Western blotting. (B) The rest cell lysate was used to extract RNA, followed by RT-qPCR measurement of the RNA levels. Relative IMP1 protein levels were quantified with the Image J software and normalized with the β-actin levels. Translational efficiency was calculated as relative protein level divided by mRNA level. Fold repression was calculated as the value in the presence of the control siRNA divided by that in the presence of the targeting siRNA. Data presented are means ± SD of three independent experiments. The *P* value is determined by two-tailed Student’s *t* test. ns, nonsignificant. *P < 0.05; **P < 0.01; ***P < 0.005. Ctrli, control siRNA; TNi, siRNAs targeting TNRC6A and TNRC6B; 4E2i, siRNA targeting eIF4E2; 4Ei, siRNA targeting eIF4E1

To further demonstrate the role of eIF4E2 in miRNA-mediated translational repression, we analyzed the effects of eIF4E2 downregulation on the expression of endogenous IMP1 in HeLa cells wherein let-7a is highly expressed. In the 3′UTR of IMP1 coding mRNA, there are six putative let-7 binding sites, which render the mRNA sensitive to the endogenous let-7 (Boyerinas et al., 2008). The IMP1 protein levels were assayed in the absence or presence of the siRNAs targeting eIF4E2. Considering that the expression level of TNRC6C is relatively low in HeLa cells compared to TNRC6A and TNRC6B (Yao et al., 2011), only TNRC6A and TNRC6B were downregulated using a mixture of siRNAs (TNi) targeting each to serve as a positive control. To determine whether eIF4E2 downregulation had any effect on IMP1 mRNA, the mRNA levels were measured. Relative translational efficiency was calculated as the protein level divided by the mRNA level. Data showed that downregulation of TNRC6A and TNRC6B increased IMP1 protein level by about 3-fold, through increasing both the mRNA level and translational efficiency (Fig. 5A and 5B). Downregulation of eIF4E2 with the two siRNAs increased IMP1 protein levels by about 6-fold and 4-fold, respectively, with little effect on the mRNA levels (Fig. 5A and 5B). In contrast, downregulation of eIF4E1 had little effect on either protein or mRNA levels (Fig. 5A and 5B). Noticeably, downregulation of TNRC6A and TNRC6B increased IMP1 protein levels by a lower magnitude compared with eIF4E2 (Fig. 5A and 5B). If RISC recruits eIF4E2 through TNRC6A to repress translation, one would expect that TNRC6A downregulation should have a more dramatic effect than eIF4E2 downregulation. We speculated that this could be accounted for by that the siRNAs targeting TNRC6A and TNRC6B were not as effective as the ones targeting eIF4E2 (Fig. 5A). Alternatively, eIF4E2 might also work through a yet identified factor to repress translation. In addition to the endogenous IMP1, downregulation of eIF4E2 increased the protein levels of endogenous PDCD4 and PTEN (Fig. S2), whose expressions are repressed by endogenous miR-21 (Asangani et al., 2008; Meng et al., 2007; Qi et al., 2009). Collectively, these results indicate that eIF4E2 is required for miRNA-mediated translational repression of endogenous proteins.

### miRNA silencing enhances eIF4E2 association with target mRNA

**Figure 6:**
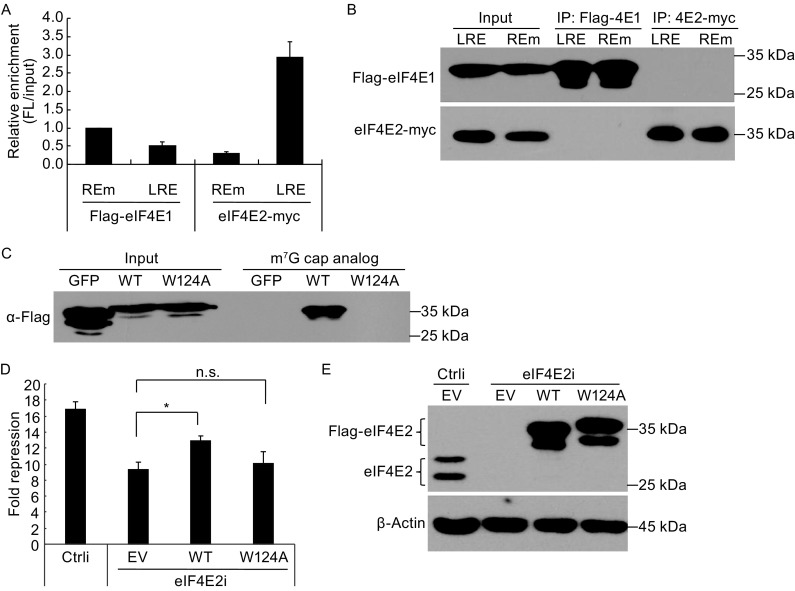
MiRNA silencing increases eIF4E2 association with target mRNA. (A and B) HEK293 cells were transfected with plasmids expressing Flag-tagged eIF4E1, myc-tagged eIF4E2, Let-7a, and reporter pLRE-fLuc or pREm-fLuc. The reporter phRL-CMV was included to serve as a control for transfection efficiency and sample handling. At 48 h postinfection, cells were lysed. (A) The cell lysate was immunoprecipitated with the anti-Flag or anti-myc affinity gel. Reporter mRNA levels were measured by RT-qPCR. The fLuc mRNA levels were normalized with the RL mRNA levels. Relative enrichment was calculated as the amount of the reporter mRNA in the precipitates divided by that in the input cell lysate. The relative enrichment of fLuc-REm mRNA by eIF4E1 was set as 1. Data presented are means ± SD of two independent experiments. (B) The cell lysate and the remaining precipitates were resolved on SDS-PAGE followed by Western blotting. (C) Flag-tagged GFP, eIF4E2 or its mutant eIF4E2-W124A was transiently expressed in HEK293T cells and pulled down with beads conjugated to the cap analog M7-GTP. The cell lysates and precipitates were analyzed by Western blotting. (D and E) HeLa cells were transfected with plasmids expressing the shRNA targeting eIF4E2, pLRE-fLuc or pREm-fLuc reporter and a renilla luciferase-expressing control reporter, and a rescue plasmid expressing the wild-type or mutant eIF4E2. At 48 h postinfection, cells were lysed. (D) Luciferase activities were measured. Firefly luciferase activity was normalized with the renilla luciferase activity. Fold repression was calculated as the normalized luciferase activity expressed from pREm-fLuc divided by that from pLRE-fLuc. Data presented are means ± SD of three independent experiments. The *P* value is determined by two-tailed Student’s *t* test. ns, nonsignificant. *P < 0.05. (E) A fraction of the cell lysate was subjected to SDS-PAGE followed by Western blotting

The affinity of eIF4E2 for the cap is about 100-fold lower than eIF4E1 (Zuberek et al., 2007), and the abundance of eIF4E2 in mammalian cells is about 10-fold lower than eIF4E1 (Kubacka et al., 2013). However, eIF4E2 can be brought to the cap by a transacting factor(s) to increase its local concentrations in the proximity of the cap of target mRNA. Based on the above results, we reasoned that miRNA on a target mRNA would increase eIF4E2 binding to the mRNA. To test this hypothesis, we analyzed the association of eIF4E2 with reporter mRNAs with or without miRNA silencing. The LRE reporter was transiently expressed in HEK293 cells, together with ectopic expression of let-7a, and Flag-tagged eIF4E1, and myc-tagged eIF4E2. The REm reporter was used as a negative control. Tagged eIF4E2 or eIF4E1 was immunoprecipitated and the amount of associated reporter mRNA was measured. Association of the protein with reporter mRNA was indicated by relative enrichment, which was calculated as the amount of precipitated RNA divided by the amount of the reporter RNA in the input. Data showed that the relative enrichment of eIF4E2 for the LRE reporter was about 10-fold higher than for the REm reporter (Fig. 6A and 6B). In contrast, the relative enrichment of eIF4E1 for the LRE reporter was modestly but significantly lower than the REm reporter (Fig. 6A and 6B). These results indicated that miRNA silencing increased the association of the target mRNA with eIF4E2 but decreased the association with eIF4E1.

These results suggested that miRNA recruited eIF4E2 to compete with eIF4E1 for binding to the target mRNA. To substantiate this notion, we analyzed whether the cap binding activity of eIF4E2 is required for its function in miRNA silencing. The residue W124 of eIF4E2 has been reported to be critical for its cap binding activity, and substitution of this residue with alanine (W124A) abolished its cap binding activity (Rom et al., 1998). The endogenous eIF4E2 was downregulated in HeLa cells. In the meanwhile, the cells were transfected with a rescue plasmid expressing either the wild-type or the mutant eIF4E2, which cannot be targeted by the siRNA. As reported previously, the mutant eIF4E2 lost the cap binding activity (Fig. 6C). Downregulation of endogenous eIF4E2 in HeLa cells reduced miRNA repression of the LRE-fLuc reporter and ectopic expression of the wild-type eIF4E2 restored the repression to some extent (Fig. 6D). In contrast, ectopic expression of the mutant eIF4E2-W124A had little effect (Fig. 6D). Downregulation of the endogenous eIF4E2 and ectopic expression of the wild-type or mutant eIF4E2 were confirmed by Western blotting (Fig. 6E). These results indicated that the cap binding affinity is required for eIF4E2 to function in miRNA silencing.

## Discussion

There is increasing evidence indicating that translation initiation is the major target of miRNA repression. It has been recently well established that miRNAs induce the dissociation of eIF4A from target mRNA, resulting in the block of the assembly of translational initiation complex (Fukaya et al., 2014; Fukao et al., 2014). However, this is unlikely the only mechanism by which miRNAs repress translation initiation. It has been extensively reported that the 5′ cap of target mRNA is important for miRNA repression (Pillai et al., 2005; Mathonnet et al., 2007; Humphreys et al. 2005; Walters et al., 2010). In this report, we compared the responses to miRNA repression of reporters with or without a structured 5′UTR. Those with a structured 5′UTR are presumably affected by eIF4A, which is an RNA helicase. We also compared reporters with or without a cap structure. The results collectively confirmed that both the cap and 5′UTR are targets of miRNA repression (Fig. 1).

We show that human TNRC6A and 6B, homologues of fly GW182, interacted with eIF4E2 (Fig. 2). We proposed that TNRC6 proteins recruited eIF4E2 to target mRNA to block translation initiation through competing with eIF4E1. This notion was supported by the observation that miRNA repression increased the association of target mRNA with eIF4E2 but decreased the association of target mRNA with eIF4E1 (Fig. 6). This notion was further supported by the observation that the cap-binding activity of eIF4E2 was required for optimal miRNA repression (Fig. 6). In order to distinguish eIF4E2 contribution to translational repression from mRNA decay in miRNA silencing, we measured reporters’ mRNA levels and analyzed the polysome profiling (Fig. 4). The results demonstrate that eIF4E2 mainly represses reporter expression on the translational level, without significantly affecting the encoding mRNA levels.

The role of eIF4E2 in miRNA repression was demonstrated by the observation that downregulation of eIF4E2 reduced miRNA repression of two different reporters (Fig. 3). We further showed that eIF4E2 downregulation increased the protein levels of endogenous IMP1, PTEN and PDCD4, whose expression are regulated by endogenous miRNAs including let-7 and miR-21 (Figs. 5 and S2).

**Figure 7:**
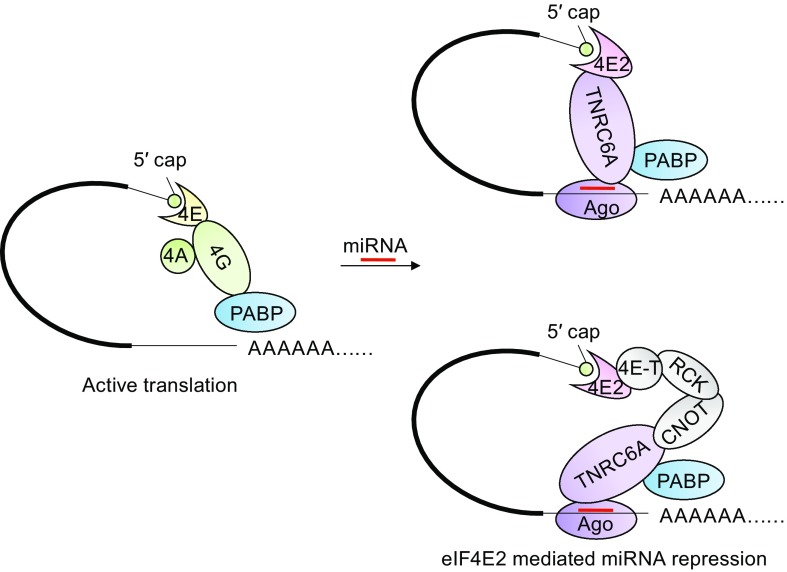
Working models for eIF4E2-mediated miRNA repression of target mRNA translation. In the absence of miRNA, target mRNA is translated actively as a 5′ cap-eIF4E-eIF4G-PABP-3′ poly(A) closed loop (left). In the presence of miRNA, RISC is loaded on the target mRNA, and eIF4E2 is recruited to the 5′ cap through direct interaction with TNRC6A (upper-right) or through the CNOT-RCK-4E-T axis (bottom-right) to compete with eIF4E, resulting in translational repression

During the course of this work, Chapat et al. reported that eIF4E2 participates in miRNA repression of target mRNA translation. They showed that miRNA recruited eIF4E2 through the CNOT1-RCK-4E-T axis. CNOT1, scaffold subunit of CCR4-NOT, recruits RCK and then 4E-T, which interacts with eIF4E2 (Chapat, 2017) (Fig. 7). Here we confirmed their result that eIF4E2 is required for miRNA repression of target mRNA translation. However, the underlying mechanism reported here is different from theirs. Here we show that eIF4E2 is recruited by miRNA through its direct interaction with TNRC6 proteins. Notably, these two mechanisms are complementary to each. Which mechanism plays a major role awaits further investigation.

## Materials and methods

### DNA constructs and siRNAs

To generate reporters pLRE-fLuc, p19RE-fLuc and pREm-fLuc, the coding sequence of renilla luciferase of phRL-CMV (Promega) was first replaced with the coding sequence of firefly luciferase (fLuc). The CMV promoter in the vector was replaced with the PGK promoter. An intron sequence of 137 bp was inserted into the 5′UTR of the reporters such that DNA contamination was excluded in RT-qPCR (Tao and Gao, 2015). Four copies of 2*LRE, 2*19RE or 2*REm were cloned into the 3′UTR of the reporters using restriction sites *Xba*I and *Not*I. Fragments of 2*LRE, 2*19RE, and 2*REm were generated by annealing paired oligonucleotides. The sequences of the oligonucleotides are listed in Table S1.

To generate mRNA reporters cap-CAA-fLuc and cap-SL-fLuc, the coding sequence and 3′UTR of pLRE-fLuc/pREm-fLuc were PCR-amplified using forward primers in which the T7 promoter and the 5′UTR (SL or CAA) sequences were built, and cloned into pMD18-T (Takara). The primer sequences are listed in Table S1.

To generate mRNA reporters EMCV-CAA-fLuc, IRES-SL-fLuc and IRES-RL, the EMCV IRES fragment PCR-amplified from pLet7-EMCV (Meijer et al., 2013) was first cloned before the coding sequence of pLRE-fLuc/pREm-fLuc/phRL-CMV using restriction sites *Sac*I and *Sac*II. CAA or SL sequences were generated by annealing paired oligonucleotides and inserted between the IRES and the firefly luciferase coding sequence using restriction sites *Sac*II and *Nco*I. The cassette covering IRES-CAA/SL-fLuc-LRE/REm was cloned into pBluescript-SK2 (-) (Stratagene) using restriction sites *Xho*I and *Bam*HI. Sequences of the primers and oligonucleotides are listed in Table S1.

The shRNAs targeting eIF4E2 and a control shRNA have been described previously (Tao and Gao, 2015). To downregulate TNRC6A and TNRC6B, a mixture of siRNAs targeting each of them were used. The sequences of the siRNAs targeting eIF4E2, eIF4E1, TNRC6A, and TNRC6B are listed in Table S1

### *In vitro* transcription

To generate mRNA reporters, the plasmids were linearized with *Bam*HI followed by chloroform extraction. The mRNAs were generated using the *in vitro* transcription system (P1300, Promega). Ribo m7G cap analog (P1711, Promega) was added to generate capped RNA transcripts. Then the produced RNA transcripts were added a poly(A) tail using the Poly(A) Tailing Kit (AM1350, Ambion).

### Cell culture and transfection

HeLa, HEK293, and HEK293T cells were maintained in Dulbecco’s modified Eagle’s medium (Invitrogen) supplemented with 10% fetal bovine serum (Gibco). DNA was transfected using Lipofectamine 2000 (Invitrogen) for HeLa cells and using Neofectin DNA transfection reagent (SciLight) for HEK293 and HEK293T cells. siRNA and mRNA reporters were transfected using Lipofectamine 2000.

### Antibodies

All the antibodies used in this report were commercially purchased: eIF4E1 (Santa Cruz, A-10, sc-271480), eIF4E2 (CST, D54C2), TNRC6A (Novus Biologicals, NBP1-28751), β-actin (GSGB-BIO, TA-09), IMP1 (Santa Cruz, sc-21026), PDCD4 (CST, D29C6), PTEN (Santa Cruz, A2B1, sc-7974), myc-specific mouse monoclonal antibody 9E10 (Santa Cruz Biotechnology, catalogue No. SC-40), Flag-specific mouse monoclonal antibody M2 (Sigma-Aldrich, catalogue No. F3165), Anti-Flag M2 affinity gel (Sigma), Anti-c-Myc Agarose (Sigma), M7-GTP-Sepharose (GE Healthcare Bioscience).

### Immunoprecipitation assay

Cells in a 60-mm dish were lysed in 500 μL Co-IP buffer (30 mmol/L Hepes, pH 7.5, 100mmol/L NaCl, 0.5% NP40), with protease inhibitor cocktail (Roche) and RNase A (sigma). The lysate was clarified at 12,000 rpm for 10 min at 4°C. 50 μL cell lysate was solved with SDS loading buffer and used as the input (10%), and the left was incubated with antibodies and protein G beads for 2–4 h at 4°C. Then the beads were washed 5 times by TBST buffer (20 mmol/L Tris-HCl, pH 7.5, 500 mmol/L NaCl, 0.1% Tween 20), and analyzed by Western Blotting.

### Pull-down assay

TNRC6A-C and Flag-tagged eIF4E/4E2 were cloned into the prokaryotic expression vector pGEX-5x-3-linker (modified from pGEX-5x-3, GE). GST-6A-C and GST-Flag-4E/4E2 were expressed in *Escherichia coli* (BL21 (DE3), Transgene) and purified following the handbook for the glutathione S-transferase (GST) fusion protein system (Amersham Biosciences). GST was removed using PreScission protease to produce Flag-4E/4E2, which was then incubated with Glutathione Sepharose 4B bound fusion protein of GST and the C-terminal domain of TNRC6A (6A-C). The precipitates were washed by TBST and resolved on SDS-PAGE followed by commassie brilliant blue staining and Western blotting.

### RNA extraction and quantitative PCR

Cytoplasmic RNA was extracted from clarified cell lysate using TRIzol (Invitrogen) following the manufacturer’s instructions without DNase treating. RNA concentration was quantified using Nanodrop (Nanodrop Technologies). 1~2 μg RNA was reverse transcribed using random primers (Takara, 3802) and MLV reverse transcriptase. No RT (minus reverse transcriptase) was included as a negative control to exclude the proper effect from plasmids DNA or genomic DNA. Quantitative RT-PCR reactions were performed using specific primers and RealMaster Mix (SYBR Green) (TIANGEN Biotech) in Corbett 6200/6600/65H0 (Corbett research) with the following cycling condition: 95°C, 10 min; 40× (95°C,10 s; 60°C, 15 s; 72°C, 20 s). The fold change on mRNA levels was calculated relative to the control and normalized to GAPDH mRNA level. Data are means of duplicate measurements by two times of 2 independent experiments. The qPCR primers for pLRE-fLuc, pREm-fLuc, and phRL-CMV have been described previously (Tao and Gao, 2015). Other primer sequences are listed in Table S1.

### Polysome profiling assay

Cells were lysed with RNC buffer (50 mmol/L Hepes, pH 7.4; 100 mmol/L KAc; 5 mmol/L MgCl_2_; 0.1% TritonX-100; 1000 U/mL RNase Inhibitor and 100 μg/mL Cycloheximide were added before using). 0.8 mL cell lysate supernatant were loaded on 12 mL 10%–50% sucrose continuous gradient followed by 36,000 rpm ultra-centrifugation for 3 h and 20 min. Then the samples were through a continuous 254-nm absorbance detector and 12 fractions, 1 mL each, were collected for each sample. 300 μL of each fraction was used for RNA extraction with 1 mL Trizol and 0.1 μg NS1 mRNA as the external reference. The fLuc-LRE and RL mRNA levels were measured by RT-qPCR normalized by NS1 level.

### RNA binding assay

Cells were lysed with cell lysis buffer (20 mmol/L HEPES KOH (pH 7.3), 150 mmol/L KCl, 10 mmol/L MgCl_2_ and 1% NP40) (Heiman et al., 2014) supplemented with the protease inhibitor cocktail (Roche), 1 mmol/L DTT, 1000 U/mL RNase inhibitor, and 30 mmol/L NaBH_3_CN (Sonenberg and Shatkin, 1977) for 30 min on a roller at 4°C. The lysate was clarified by centrifugation at 12,000 rpm for 15 min at 4°C. The clarified lysate was incubated with Anti-c-Myc Agarose (Sigma) and Anti-Flag M2 affinity gel (Sigma) separately for 2 h on a roller at 4°C. Immunoprecipitates were washed three times with TBST. Ten percents of the immunoprecipitate was resuspended in SDS loading buffer for protein detection by Western blotting. The remaining immunoprecipitate was resuspended in TRIzol (Invitrogen) for RNA extraction.

## Electronic supplementary material

Below is the link to the electronic supplementary material.

Supplementary material 1 (PDF 258 kb)
