# Supplementary material for: MicroRNAs recruit eIF4E2 to repress translation of target mRNAs
Source: Protein Cell. 2017 Jul 28;8(10):750–61. doi: 10.1007/s13238-017-0444-0 (PMC5636748; doi:10.1007/s13238-017-0444-0)
Supplement: Supplementary file 1 — Supplementary material 1 (PDF 258 kb) [file 13238_2017_444_MOESM1_ESM.pdf]

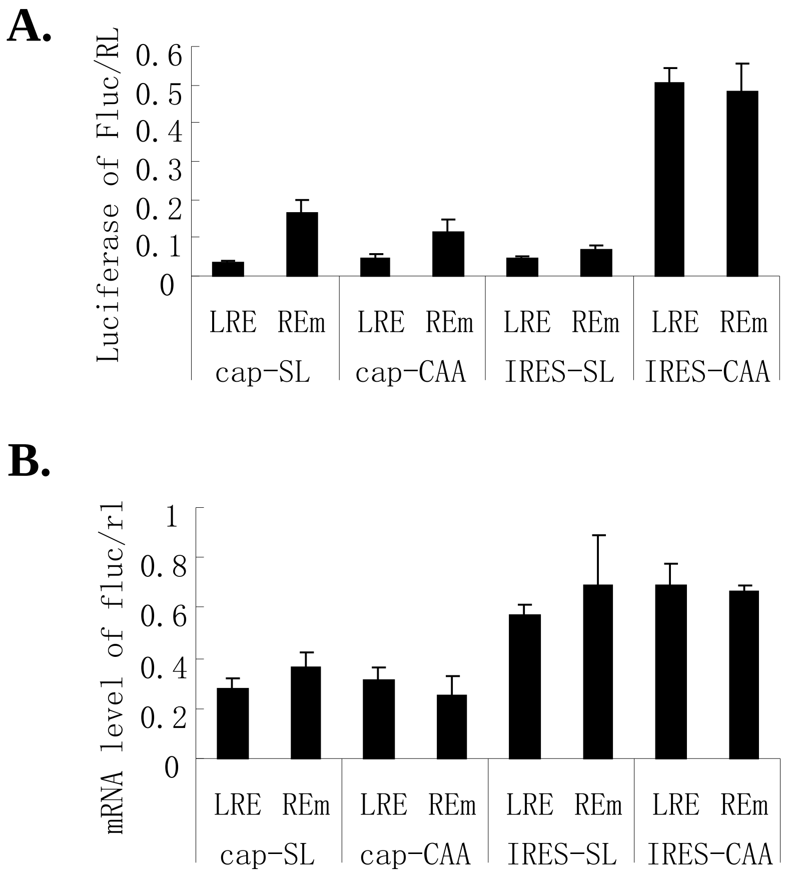

Figure S1. Luciferase and mRNA level of reporters in Figure 1.

(A) Data from Figure 1B are represented as relative luciferase Fluc/RL. (B) Lysate in Figure 1B were quantified by RT-qPCR after RNA extraction and reverse transcription. Y-axis represents the relative mRNA level fluc/ rl. Data presented are means ± SD of three independent experiments.

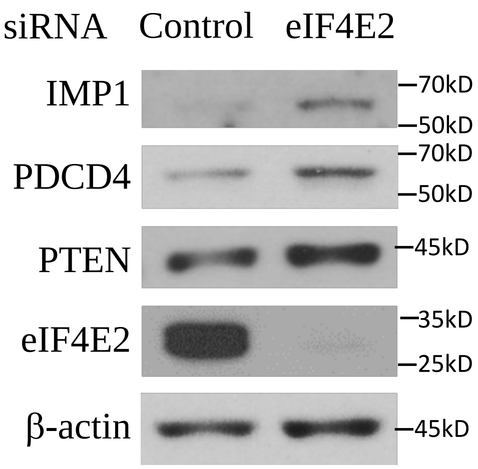

Figure S2. Downregulation of eIF4E2 increases the protein levels of endogenous IMP1, PDCD4 and PTEN.

HeLa cells were transfected with siRNAs indicated. At 36h posttransfection, cells were lysed and subjected to SDS-PAGE followed by Western blotting.

Oligos for generating plasmids reporters	
**primer**	**sequence**
2*LRE-sense	5’-ctagtactatacaataggctacctcatctagaatcggcggccgcactatacaataggctacctcacg-3’
2*LRE-antisense	5’-ggcccgtgaggtagcctattgtatagtgcggccgccgattctagatgaggtagcctattgtatagta-3’
2*19RE-sense	5’-ctagtagttttgcatccatttgcacatctagaatcggcggccgcagttttgcatccatttgcacacg-3’
2*19RE-antisense	5’-ggcccgtgtgcaaatggatgcaaaactgcggccgccgattctagatgtgcaaatggatgcaaaacta-3’
2*REm-sense	5’-ctagtgaccacgcttatgacatactcctctagaatcggcggccgcgaccacgcttatgacatactcccg-3’
2*REm-antisense	5’-ggcccgggagtatgtcataagcgtggtcgcggccgccgattctagaggagtatgtcataagcgtggtca-3’
**Primers for generating mRNA reporters**	
T7-CAA-CDS-Forward	5'-gtaatacgactcactatagggcaacaacaacaacaacaacaacaacaacaacaacaacaacaa caacaacaacaacaacaagccaccatggaagacgccaa-3'
T7- SL-CDS-Forward	5'-gtaatacgactcactatagggcaacaacaaacatttgcttctgacacaactgtgttcactagcaacctcaaa cagacaccatgacgccaccatggaagacgccaa-3'
fLuc-Reverse	5'-ttatcgattttaccacattt-3'
EMCV-Sac I-Forward	5'-gcacgagctcCCTCGAGCGGGATCAATTCC-3'
EMCV-Sac II-Reverse	5'-gcacccgcggattatcgtgtttttcaaa-3'
**Oligos for generating mRNA reporters**	
SL-sense	5'-ggacatttgcttctgacacaactgtgttcactagcaacctcaaacagacaccatgacc-3'
SL-antisense	5'-catgggtcatggtgtctgtttgaggttgctagtgaacacagttgtgtcagaagcaaatgtccgc-3'
CAA-sense	5'-ggcaacaacaacaacaacaacaacaacaacaacaacaacaacaacaacaacaacaagccac-3'
CAA-antisense	5'-catggtggcttgttgttgttgttgttgttgttgttgttgttgttgttgttgttgttgccgc-3'
**siRNA oligos**	
si4E2-1	5’-CACAGAGCTATGAACAGAATA-3’
si4E2-2	5’-CUCACACCGACAGCAUCAA-3’
si4E1	5’-GGAUGGUAUUGAGCCUAUG-3’
siTNRC6A	5’-GCCUAAAUAUUGGUGAUUA-3’
siTNRC6B	5’-GCACTGCCCTGATCCGATA-3’
**Primers for RT-qPCR**	
fLuc-Forward	5’-CCAGGGATTTCAGTCGATGT-3’
fLuc-Reverse	5’-AATCTcACGCAGGCAGTTCT-3’
RL-Forward	5’-TGAGGAGTTCGCTGCCTACC-3’
RL-Reverse	5’-TGCGGACAATCTGGACGACG-3’
IMP1-Forward	5’-GGCCATCGAGAATTGTTGCAG-3’
IMP1-Reverse	5’-CCAGGGATCAGGTGAGACTG-3’
GAPDH-Forward	5’-TCGGAGTCAACGGATTTG-3’
GAPDH-Reverse	5’-GCATCGCCCCACTTGATT-3’

Table S1. Oligonucleotide sequences used in cloning and RT-qPCR.
